# Capturing causal claims: A fine-tuned text mining model for extracting causal sentences from social science papers

**DOI:** 10.1017/rsm.2024.13

**Published:** 2025-03-10

**Authors:** Rasoul Norouzi, Bennett Kleinberg, Jeroen K. Vermunt, Caspar J. van Lissa

**Affiliations:** 1 Methodology and Statistics, Tilburg University, Tilburg, Netherlands; 2 Department of Security and Crime Science, University College London, London, UK

**Keywords:** causal sentences dataset, causal text mining, domain-specific fine-tuning, social science, transformer models

## Abstract

Understanding causality is crucial for social scientific research to develop strong theories and inform practice. However, explicit discussion of causality is often lacking in social science literature due to ambiguous causal language. This paper introduces a text mining model fine-tuned to extract causal sentences from full-text social science papers. A dataset of 529 causal and 529 non-causal sentences manually annotated from the Cooperation Databank (CoDa) was curated to train and evaluate the model. Several pre-trained language models (BERT, SciBERT, RoBERTa, LLAMA, and Mistral) were fine-tuned on this dataset and general-purpose causality datasets. Model performance was evaluated on held-out social science and general-purpose test sets. Results showed that fine-tuning transformer models on the social science dataset significantly improved causal sentence extraction, even with limited data, compared to the models fine-tuned only on the general-purpose data. Results indicate the importance of domain-specific fine-tuning and data for accurately capturing causal language in academic writing. This automated causal sentence extraction method enables comprehensive, large-scale analysis of causal claims across the social sciences. By systematically cataloging existing causal statements, this work lays the foundation for further research to uncover the mechanisms underlying social phenomena, inform theory development, and strengthen the methodological rigor of the field.

## Highlights

**What is already known:**

- Causality is crucial for theory development.
- Causality is not always explicit in social science text.
- Existing causal extraction methods lack social science specificity.

**What is new:**

- Introduced a model fine-tuned for social science causality extraction.
- Curated a social science-specific causal sentence dataset.

**Potential impact:**

- Enables large-scale causal claim analysis.
- Aids in theory development and practice.

## 1 Introduction

Understanding causality is central to pursuing scientific knowledge, including in the social sciences.^1^ Causal explanations are a hallmark of “strong theories” that go beyond mere descriptions of empirical patterns, elucidating the underlying mechanisms.^2^^,^ ^3^ Moreover, causality is a necessary assumption when using research findings to inform treatments, interventions, and decisions in policy, parenting, or clinical practice.^4^ However, the social sciences have had an uncomfortable relationship with causality since the early 1900s,^1^ which is still evident today.^5^ The central problem is that causality is not always explicitly discussed.^6^ This vagueness and “strategic ambiguity” about causality has likely contributed to concerns over a “theory crisis” in the social sciences.^7^ Causal assumptions play a crucial role in substantiating scientific hypotheses, so ambiguity about causality impedes the proper functioning of the scientific method and, ultimately, the advancement of our understanding of social scientific phenomena.^8^^,^ ^9^ Given their significance for theory and practice, much can be gained by making an inventory of causal claims in the published social scientific literature. An overview of existing causal claims can inform theory development, justify practical applications, and strengthen methodological foundations guiding future theory-testing research.

Causal claims extraction is part of the emerging field of text mining systematic review methods; a collection of qualitative research synthesis methods aided by quantitative text mining and natural language processing methods. Text mining techniques can inductively identify important themes and relationships within a corpus of literature.^10^ Currently, some methods are available to automatically extract causal claims from scientific text.^11^ However, a significant shortcoming of existing techniques is that none have been developed with attention to the idiosyncrasies of social scientific writing. These idiosyncrasies include using nuanced and ambiguous language when discussing causality, the tendency to employ hedging or tentative statements about causal relationships, and the statement of causal claims as implicit rather than explicit.^12^ Recognizing the need for a method that encompasses the full spectrum of social science discourse, it becomes imperative to analyze causal statements across the entirety of a paper. This comprehensive perspective is essential, as causality can manifest in various sections, from introduction to conclusion, a consideration not addressed by current methodologies. To fill this gap, this paper introduces a text-mining model that extracts causal claims from full-text papers. The model intends to extract and catalog causal statements in social science literature, providing a tool to automate coding for systematic reviews of causal claims.

This study set out to evaluate the ability of several candidate models to classify causal and non-causal sentences in social science literature. All models were fine-tuned on a general-purpose training dataset, and a newly curated dataset tailored specifically to the domain of social science, comprising 529 causal and 529 non-causal sentences extracted from the Cooperation Databank (CoDa); a comprehensive annotated archive of all studies on human cooperation.^13^ The study design was a 5 (Model: BERT, Sibert, Roberta Large, LLAMA2-7b, Mistral-7b) x 3 (Training data: general-purpose, social science, general-purpose and social science) x 2 (Testing data: general-purpose, social science) factorial design. This allows us to investigate how well the different models perform, as well as determine the added value of fine-tuning these models on social scientific data above and beyond a general-purpose dataset.

This paper makes two contributions to the field of causal sentence extraction in social sciences. First, we present a new, manually curated dataset of causal sentences drawn from social science literature. This dataset complements existing general-purpose datasets by capturing the unique linguistic patterns and nuances of causal claims in social science texts. This dataset, after splitting into training and test sets, is used for fine-tuning and testing candidate models. Second, we introduce causality extraction models that have been finetuned on this novel social science dataset. Our approach addresses the challenges posed by the often-ambiguous language used in social science literature to express causality, thereby enhancing the accuracy and applicability of automated causal extraction in this domain. Furthermore, the annotated data and tuned models serve as a foundation for further advancements in extracting and analyzing causal sentences from the vast body of social science literature.

We address the following research questions:

- Which model performs best when extracting causal language sentences from social science texts?
- Is there evidence for a domain shift bias?
- Does fine-tuning on a domain-specific dataset increase performance?

Open Science Statement: We openly share the dataset and code underpinning this research at https://github.com/rasoulnorouzi/cessc. The repository also includes a tutorial on the setup and execution process for ease of use and reproducibility. Both the dataset and code are released under the GNU GPLv3 license, which encourages use and modification with appropriate attribution.

## 2 Literature review

### 2.1 Causality extraction in social science

The extraction of causal relations from unstructured text poses significant challenges due to the complexity and variability of human language, as well as the diverse patterns used to represent causality. Causal language involves clauses or phrases where one event, state, action, or entity is explicitly portrayed as influencing another. Linguistic cues of causation abound, with causative verbs (such as “increase,” “decrease,” or “improve”) often denoting a causal relationship. Conjunctions such as “because,” “due to,” and “since” are commonly used to express causality.^12^ Causal language is, to some extent, domain specific. As a result, causal sentence extraction techniques developed in one scientific field often perform worse when applied in other fields; a phenomenon known as domain shift bias.^14^ This is especially the case in the social sciences, where domain shift bias is intensified due to the historical reluctance to employ explicit causal language,^5^ a trend not commonly observed in other disciplines. This hesitation leads to vague and indirect expressions about causality, complicating the task of identifying causal connections. For instance, terms like “explain,” “influence,” and “predict,” suggest causality but maintain some ambiguity about the actual nature of the relationships involved. This vagueness is a significant hurdle for causal sentence extraction. This ambiguity is further compounded by the use of hedging language in scientific writing, which introduces uncertainty and increases the risk of misinterpretation or misrepresentation of the causal links being studied. This practice not only impedes clear understanding but also hampers the advancement of knowledge in these fields, as it fosters a culture of “truthiness,” where personal beliefs or assumptions are presented as facts without adequate evidence.^15^^,^ ^16^

Prior work has studied quantitative differences in writing styles between social science and other disciplines. Results showed that sentences in social scientific writing were, on average, longer (28.62 words in Anthropology papers versus 21.37 words in Electrical Engineering papers^17^) and contained more clauses per sentence (2.69 in Educational Administration papers compared to 2.38 in Communicable Diseases papers^18^). Taken together, these differences lead to more complex syntactic structures. Social science papers more often used relative clauses (29.2% in Educational Administration papers versus 20.5% in Air Pollution papers), active voice constructions (77.9% in Educational Administration papers compared to 65.5% in Air Pollution papers^18^), and finite dependent clauses (0.79 in anthropological papers versus 0.37 in electrical engineering papers). Social sciences also showed higher left-embeddedness in sentences—a pattern of writing where the main verb (“showed” in this sentence) comes relatively late in a sentence (the average number of words before the main verb was M = 7.88 in anthropology, versus M = 6.37 words in electrical engineering^17^). Social sciences tend to use more nominal expressions of causality with complex syntactic structures, such as “reason,” “cause,” and “implication,” whereas hard sciences use simpler prepositions and conjunctions to signify causal relationships, such as “since,” “because,” and “because of,” In sum, writing in the social sciences tends to be less structured and more implicit style, with a greater role for the voice of the author.^19^ Writing in the hard sciences, by contrast, tends to be more formally structured and patterned, with a more objective and compact writing style.

### 2.2 Prior work in causality extraction

Existing techniques for causal claims extraction can be broadly categorized into three main groups: knowledge-based methods, statistical machine learning methods, and deep learning methods.^11^ Knowledge-based methods rely on manually constructed rules, patterns, and domain expertise.^20^ These methods use predefined patterns, such as “X causes Y” or “Y because of X,” to identify causal relationships in a text. For example, in the sentence “Smoking causes lung cancer” a knowledge-based system would recognize “causes” as a causal indicator linking “smoking” to “lung cancer.” Although knowledge-based methods demonstrate suitability for extracting causal relationships within individual sentences, they require considerable human effort and encounter challenges in formulating comprehensive rules and patterns to discern the nuanced connections between words. Moreover, the generalizability of these methods is constrained by the restricted scope of the training data upon which they are developed.^11^

Machine learning methods use natural language processing (NLP) techniques, which are computational approaches to understanding and manipulating human language, and annotated corpora, collections of text labeled with causal information. These methods extract relevant features from the text, which are then used to train machine learning algorithms to identify causal relationships.^21^ For example, these algorithms might learn to recognize words like “because” or “therefore” as indicators of causality. These methods offer more automation than knowledge-based approaches but still require labor-intensive feature engineering to identify the text’s most relevant aspects for detecting causality. This might involve selecting specific words, grammatical structures, or sentence patterns that are indicative of causal relationships.^11^

The third category comprises deep learning methods, which automatically learn condensed numerical representations of words, known as word embeddings. These embeddings capture semantic relationships, allowing the model to understand that words like “cause,” “lead to,” and “result in” have similar meanings in the context of causality. This allows for better representation learning and cross-domain portability.^11^ Recurrent neural networks, convolutional neural networks, and graph convolutional networks are common deep learning models for causal relation extraction. Recurrent neural networks process text sequentially, helping them better understand sentence context. Convolutional neural networks identify patterns in text similar to how they detect patterns in images, allowing them to recognize causal patterns regardless of their position in a sentence. Graph convolutional networks model relationships between different parts of a sentence, potentially capturing complex causal structures.^22^

In the development of NLP techniques, the advent of BERT (Bidirectional Encoder Representations from Transformers) introduced a paradigm shift toward models equipped with transformer mechanisms. Transformers are a type of neural network architecture that can consider the entire context of a sentence when processing each word. This is crucial for understanding causality, as the causal meaning of a word often depends on its context. These models, by design, take the context of words within sentences into account, showcasing a marked improvement over prior methodologies.^23^ For instance, BERT can distinguish between “insomnia causes anxiety” and “anxiety causes insomnia” by understanding that the causal relationship is different despite using exact words. The advancement of transformer models not only highlights the deficiencies of earlier approaches in understanding language nuances; it has also shown superior performance in benchmark evaluations, surpassing previous knowledge-based and statistical methods in the precision, the accuracy of identified causal relationships, and recall of extracted causal relationships.^24^ Recent research has further improved relation extraction through large language models (LLMs), which prove to be very efficient in extracting temporal and causal relationships from texts.^25^ Furthermore, context-enhanced transformers have shown that they improve generalization by reducing excessive dependence on specific sentence structures and allowing robust performance even with limited training data.^26^

Training transformer models requires superlative computational resources and exceedingly large corpora of training data. Consequently, most research does not train models from scratch but uses pre-trained models instead. Pre-trained models can be fine-tuned to improve their performance on specific tasks. Fine-tuning constitutes an optimization process whereby a pre-trained language model undergoes parameter adjustments tailored to a specific task, enabling the model to leverage its prior knowledge while adapting to the new data. Notably, the pre-training paradigm facilitates fine-tuning on downstream tasks using substantially smaller datasets compared to training from scratch, thereby underscoring the adaptability and effectiveness of such models across a diverse array of applications.^27^ The present study employs the fine-tuning procedure for the binary classification task, wherein the objective is to classify sentences extracted from the social science literature as either exhibiting causality or lacking causality. However, a notable concern is the substantial computational resources required, which has been somewhat alleviated by emerging techniques such as Parameter-Efficient tuning.^28^ This technique optimizes a small portion of model parameters while keeping the rest fixed, significantly reducing computation and storage costs. This development offers a promising way to harness the computational power of these models more efficiently. Given their profound understanding of context and nuanced capabilities, this study aims to employ transformer models to leverage their strengths in addressing challenges posed by limited data resources.

## 3 Method

### 3.1 Overview

Our study aimed to fine-tune and evaluate pre-trained language models for automatically identifying causal statements in social science papers. At a high level, our process involved the following steps:

1. **Data collection and preparation:** We prepared two datasets: a) A general-purpose dataset compiled from existing open-source datasets. b) A social science-specific dataset curated from full-text papers in the Co. Both datasets contained sentences labeled as causal or non-causal.
2. **Model selection:** We chose several pre-existing language models for fine-tuning.
3. **Fine-tuning:** We adapted these models to the task of causal sentence extraction using our prepared datasets.
4. **Model evaluation:** We assessed how well different fine-tuned models performed this classification task.

In the following, we detail each step of this process, the specific models we used, how we fine-tuned them, and how we evaluated their performance.

### 3.2 Datasets

#### 3.2.1 General purpose dataset

We drew upon earlier work that introduced six datasets for analyzing causal sentences.^29^ However, as the Penn Discourse Treebank (PDTB) dataset is not openly available, it was excluded from our study. The following is a summary of the datasets that were utilized to construct the General-Purpose Dataset:

**AltLex:** Annotated causal language utilizing alternative lexicalizations within single sentences from news articles. However, it has limitations including small size, exclusion of implicit signals, and consideration solely of intra-sentence relations.

**BECAUSE 2.0:** Annotations of cause, effect, and connective spans based on Construction Grammar principles within single sentences from diverse sources. Its limitations comprise a modest size and omission of inter-sentence relations.

**CausalTimeBank (CTB):** Explicit causal relation annotations between events within the TempEval-3 corpus. It focuses solely on annotating events and disregards contextual information.

**EventStoryLine (ESL):** Annotations of both explicit and implicit causal relations between events in the Event Coreference Bank. It shares similar limitations with CTB in terms of event-centric annotation.

**SemEval 2010 Task 8:** Originally annotated for classifying semantic relations between noun phrases, with limitations including the absence of contextual argument information and annotation restricted to inter-sentence relations.

Table 1 displays the number of causal and non-causal samples for both training and validation sets of each dataset separately. We should point out that the number of samples and their balance report is before preprocessing, as there were many duplicated samples in them.

**Table 1:** Overview of five open-source datasets that combined to form the general-purpose dataset

Corpus	Split	Causal	Non-causal	Sample causal sentence
AltLex	training	326	285	In North Korea, heavy rains from the storm **caused** widespread flooding and damage.
	test	130	286	
BECAUSE 2.0	training	473	107	**Thanks to** the fast action of the Federal Reserve in cooperation with the SEC and the Treasury, we dodged a bullet when Bear Stearns collapsed.
	test	15	4	
CausalTimeBank (CTB)	Training	718	2,599	In space, some say female pilots were held up until now **by** the lack of piloting opportunities for them in the military.
	test	100	392	
EventStoryLine (ESL)	training	9,146	8,268	She is interested in going back to Betty Ford **because** she knows everyone there.
	test	894	1073	
SemEval 2010 Task 8	training	1,003	6,997	The current view is that the chronic inflammation in the distal part of the stomach **caused by** Helicobacter pylori infection results in an increased acid production from the non-infected upper corpus region of the stomach.
	test	328	2,389	

*Note*: The **bold phrases** indicate causal expressions between events or states. These are general-purpose datasets without field-specific categories. The numbers in the Causal and Non-Causal columns represent the count of sentences classified as causal or non-causal in each dataset split.

#### 3.2.2 General purpose dataset preprocessing

The training sets from the five aforementioned open-source datasets were combined to create a general-purpose training set, and the test sets from these datasets were likewise combined to create a general-purpose test set. Of the initial dataset (29,922 in training data and 5,611 in test data), we removed duplicates and retained 12,834 training and 3,679 test samples. The training set was imbalanced (9,954 were causal and 2,880 were non-causal), posing a risk of bias during model fine-tuning. This imbalance could lead to the model bias toward the majority class (non-causal sentences) during training and, consequently, can lead to poor performance in the minority class (causal sentences).^30^ To counter this, we applied an undersampling technique, resulting in a balanced general-purpose training set with 5,760 samples, equally divided between causal and non-causal sentences. This undersampling was performed by randomly removing non-causal examples (the majority class) until the training dataset was balanced. Although the test set was imbalanced, with 3,070 non-causal and 609 causal sentences, we left it as is. This decision was made to ensure that the test set accurately reflects real-world conditions, providing an ecologically valid estimate of the model’s performance. The training set was then randomly divided into 20 percent for validation and the remaining 80 percent for training. The validation set, distinct from the test set, was used during the training process to tune the model parameters and prevent over-fitting, while the test set remained untouched for the final performance evaluation. Overfitting occurs when a model becomes too specialized to its training data, learning the underlying patterns and the random fluctuations and noise specific to that dataset. This excessive adaptation leads to a model that performs exceptionally well on the training data but fails to generalize effectively to new, unseen examples.^31^ An overfitted model memorizes the training set rather than learning the true underlying relationships, resulting in poor performance when applied to real-world data outside its training experience.

#### 3.2.3 Curated social science dataset

We manually curated an additional dataset aimed at understanding the use of causal language in social science literature based on the CoDa, a comprehensive collection of all papers dedicated to game theory applications within social science from 1958–2017 across 78 societies.^13^ Like other existing benchmarks, CoDa is a convenience sample rather than a representative sample of social science literature, a common limitation due to access to full-text papers such as paywalls and a lack of standardized APIs.^10^ Although acknowledging these representation limitations, CoDa’s highly curated nature is suitable for the validation of our method.

From this dataset, 2,590 articles were converted into raw text using the Grobid library in Python and subsequently segmented at the sentence level.^32^ Following conversion, a pre-processing stage corrected common errors arising during PDF-to-text translation. These errors typically involve misinterpretations of similar characters, such as “0” and “O,” “b” and “6” or incorrect joining or splitting of letters.

One of the authors (RN) initially labeled sentences using the Doccano web annotation tool,^33^ categorizing them as causal, non-causal, or potentially ambiguous. Sentences marked as potentially ambiguous (117 of 1058 sentences; 11.05%) were subsequently reviewed by all authors to determine their final classification as either causal or non-causal. Inter-rater agreement was estimated using Fleiss’ Kappa index (Fleiss, 1971), resulting in 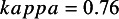 , denoting a “substantial” agreement. For cases where consensus remained elusive, A majority voting method was employed to finalize the labels for samples where consensus was elusive. Ultimately, this process resulted in a curated dataset of 529 causal and 529 non-causal sentences, with no ambiguous category in the final dataset. Table 2 presents some examples of the curated social science dataset.

**Table 2:** Examples of sentences and their corresponding labels from the final curated social science dataset

Sentence	Label
Violent media may cause viewers to perceive greater anger and less gratitude during a cooperative task and as such be less inclined to cooperate.	Causal
Even though the level of regulation a facility faces does not depend on the overall size of a facility, the two factors are highly correlated.	Non-causal
This result is consistent with a previous study which suggests that behaving dishonestly leads to the forgetting of rules.	Causal
We find differences in the behavior of our experimental participants across the three countries, and we find that Hofstede’s dimensions help to explain these differences.	Non-causal

While there is no universally optimal standard for dataset partitioning in deep learning, established practices typically allocate 70%–80% of samples to the training set, with the remainder reserved for model validation and testing. In this study, we randomly divided our balanced dataset into training (70%), validation (10%), and test (20%) sets. This partition ratio ensures sufficient samples for model training while mitigating overfitting and provides a reliable test set for performance evaluation.^34^

#### 3.2.4 Merged dataset

To create this dataset, we combined the preprocessed and balanced general-purpose training set with the social science training set. From this merged training data, we then allocated 80% for actual training and 20% for validation. The original test sets (both general-purpose and social science) were kept separate and untouched to maintain the integrity of our evaluation.

### 3.3 Candidate models

We included examples of prevalent model architectures—models using both Masked Language Model (MLM) and Causal Language Model (CLM) pre-training methods—to assess their performance on the task of causal sentence extraction. Our aim was not to directly compare these pre-training methods but to evaluate a selection of models relevant to this specific task. MLM is a type of self-supervised learning task that involves predicting masked tokens in a sequence of text. For example, given the sentence “Heavy [MASK] from the storm caused widespread flooding and damage.”, the model would try to predict the word “rains” to fill in the [MASK]. The model is trained to predict the original tokens based on the context of the surrounding words. Models like BERT, SciBERT, and RoBERTa were pre-trained using MLM. CLM, also known as a unidirectional language model, is a pre-training task that predicts the next token (e.g., word) in a sequence based on the preceding context. For instance, given the partial sentence “Heavy Rains from the storm caused widespread flooding and […],” the model might predict “damage” as the next word. In other words, the model learns to generate text in a forward direction, token by token, using the previously generated tokens as context. We selected the following models for consideration:

**BERT Model:** The architecture of this model is described in Devlin et al.,^23^ and is pre-trained by the MLM method. In prior research, this model showed good performance in terms of extracting causality in six benchmark datasets.^24^

**Science BERT(SciBERT)^35^:** The SciBERT model was pre-trained on a corpus of scientific literature, which makes it particularly well-suited to scientific text mining applications. It has not been previously used for causality extraction.

**RoBERTa large^36^:** RoBERTa is also built upon the BERT architecture but incorporates a larger number of parameters and was pre-trained on a more extensive corpus. It has demonstrated improved performance over BERT on downstream tasks such as sequence classification (analogous to this study) and question answering. Consequently, RoBERTa presents itself as a viable candidate for investigating the trade-off between foundational language models like BERT and more complex models, thereby shedding light on the potential advantages of pre-training on bigger datasets and increased model complexity.

**LLAMA 2-7b^37^ and Mistral-7b^38^:** The Mistral and LLAMA 2 models are chosen due to their state-of-the-art generative capabilities and usage of CLM for pre-training. The 7b in their name refers to the model’s seven billion parameters.

### 3.4 Models fine-tuning

The fine-tuning of our models was conducted using the Python programming language, utilizing the PyTorch framework^39^ and the Hugging Face library.^40^ We used the default hyperparameters provided by the Hugging Face documentation for all models.^41^ To mitigate potential overfitting, we implemented regularization and utilized default dropout rates. We employed a validation set to select the best model based on the F1 score at the end of the fine-tuning process. The test set remained untouched throughout this process and was used solely for final evaluation.

Given the substantial size of the LLAMA2-7b and Mistral-7b models, which contain billions of parameters, their fine-tuning presented an additional layer of complexity. These large models typically require multiple high-powered GPUs for training, which can be resource intensive. To facilitate training on a single GPU, we employed a parameter-efficient tuning method known as Quantized Low Rank Adaptation (QLoRA).^28^ This method allows us to fine-tune large models using less computational resources by adjusting only a small subset of the model’s parameters. This technique has been implemented using the bitsandbytes library,^42^ in conjunction with the Hugging Face Transformers and PEFT frameworks.

### 3.5 Evaluation metrics

The evaluation of model performance on the test sets was conducted using metrics commonly employed in classification^11^:

Precision, recall, F1-score, and a macro average F1-score. For our binary classification task, a positive prediction indicates the presence of a causal claim, while a negative prediction indicates a non-causal sentence. Precision is the number of true positive predictions (correctly identified causal sentences), divided by the total number of observations predicted as causal. Recall is the ratio of true positive predictions to the total number of true positive observations (all actual causal sentences in the dataset). Both measures are also calculated for negative observations (non-causal sentences). The F1 Score is a harmonic average of Precision and Recall and indicates whether a model has a good balance between precision and recall. Since there is a substantial imbalance between positive and negative observations in the general test data, we also computed a macro average F1 score, accounting for this imbalance by calculating the metric independently for each class and then taking the unweighted mean across classes. We use the macro average F1 score as our primary criterion for determining the ‘best’ performing model, as it provides a balanced measure of performance across both classes, regardless of their proportions in the dataset.

## 4 Results

Table 3 details the performance of each model, as measured by the predefined metrics, after training on each of the datasets. These results are presented for both the general-purpose and social science test sets.

**Table 3:** Detailed performance metrics of language models on general-purpose and social science test sets, showcasing precision, and F1 scores for causal and non-causal classes

Model name	Training set	Test set	Positive (Causal)			Negative (Non-causal)			Overall score
			Precision	Recall	F1	Precision	Recall	F1	F1 Macro avg
BERT	General	General	0.55	0.88	0.68	0.97	0.86	0.91	0.79
		Social Science	0.72	0.79	0.76	0.77	0.70	0.73	0.74
	Social Science	General	0.51	0.31	0.39	0.87	0.94	0.91	0.65
		Social Science	0.85	0.94	0.89	0.94	0.83	0.88	0.89
	Merged	General	0.51	0.90	0.65	0.98	0.83	0.90	0.77
		Social Science	0.82	0.89	0.85	0.88	0.80	0.84	0.85
SciBERT	General	General	0.55	0.84	0.67	0.97	0.86	0.91	0.79
		Social Science	0.73	0.76	0.74	0.75	0.72	0.73	0.74
	Social Science	General	0.41	0.39	0.40	0.88	0.89	0.89	0.64
		Social Science	0.91	0.85	0.88	0.86	0.91	0.89	0.88
	Merged	General	0.56	0.85	0.67	0.97	0.87	0.91	0.79
		Social Science	0.86	0.84	0.85	0.84	0.86	0.85	0.85
RobertLarge	General	General	0.58	0.87	0.70	0.97	0.87	0.92	0.81
		Social Science	0.76	0.78	0.77	0.78	0.76	0.77	0.77
	Social Science	General	0.48	0.36	0.41	0.88	0.92	0.90	0.65
		Social Science	0.85	0.91	0.88	0.90	0.84	0.87	0.87
	Merged	General	0.55	0.89	0.66	0.98	0.84	0.90	0.78
		Social Science	0.82	0.89	0.85	0.88	0.80	0.84	0.85
LLAMA2-7b	General	General	0.49	0.82	0.62	0.96	0.83	0.89	0.75
		Social Science	0.63	0.94	0.75	0.89	0.44	0.58	0.67
	Social Science	General	0.50	0.01	0.02	0.84	1.00	0.91	0.47
		Social Science	0.84	0.75	0.79	0.77	0.86	0.82	0.80
	Merged	General	0.52	0.81	0.64	0.96	0.85	0.90	0.77
		Social Science	0.87	0.84	0.85	0.84	0.87	0.86	0.85
Mistral-7b	General	General	0.49	0.85	0.66	0.97	0.83	0.89	0.78
		Social Science	0.66	0.85	0.74	0.79	0.57	0.66	0.70
	Social Science	General	0.29	0.03	0.05	0.84	0.99	0.91	0.48
		Social Science	0.77	0.77	0.77	0.77	0.76	0.77	0.77
	Merged	General	0.48	0.85	0.62	0.97	0.82	0.89	0.75
		Social Science	0.79	0.84	0.81	0.83	0.78	0.80	0.81

**RQ-a) Which model performs best when extracting causal language sentences from social science texts?**

The best-performing model for extracting causal language sentences from social science texts is BERT, fine-tuned on the social science training set. This model achieved the highest F1 macro score of 0.89 on the social science test set. A bootstrap analysis with 10,000 resamples, and a 95% confidence interval yielded an F1 score of 0.88 with a confidence interval of (0.84, 0.92) for this model when tested on the social science test set.

**RQ-b) Is there evidence for a domain shift bias?**

The models fine-tuned on the general-purpose training set exhibited a performance decline when evaluated on domain-specific social science data compared to the general-purpose test set, indicating a domain shift bias. Considering the F1 macro average, the BERT model’s performance decreased by approximately 5%, SciBERT by 5%, RobertaLarge by 4%, and LLAMA2-7b and Mistral-7b by 18% and 8%, respectively. A bootstrap hypothesis test was conducted for the BERT model, comparing its performance when fine-tuned on general-purpose data and tested on the general-purpose test set (F1 = 0.78, CI: [0.77, 0.80]) versus the social science test set (F1 = 0.74, CI: [0.68, 0.79]). The test yielded a 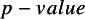 of 0.04, confirming a statistically significant decrease in performance when applied to the domain-specific data.

**RQ-c) Does fine-tuning on a domain-specific dataset increase performance?**

Models fine-tuned on the social science training set exhibited higher F1 macro average scores when evaluated on the social science test set compared to models fine-tuned on general-purpose datasets. The BERT model showed a 15% increase, SciBERT a 14% increase, RobertaLarge a 10% increase, LLAMA2-7b a 13% increase, and Mistral-7b a 7% increase. For the best-performing BERT model on social science data, a bootstrap hypothesis test was conducted to compare its performance when fine-tuned on general-purpose data (F1 = 0.73, CI: [0.68, 0.79]) versus social science data (F1 = 0.88, CI: [0.84, 0.92]). This test assesses whether the observed performance improvement is statistically significant. The results yielded a 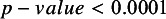 and 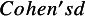 of 6.15, indicating a statistically significant improvement with a large effect size.

### 4.1 Error analysis

To assess the model’s performance on potentially challenging cases, we analyzed cases identified as ambiguous during the labeling process. We examined whether these cases were classified with lower accuracy than others by the best-performing model for the social science test set, which was BERT fine-tuned on the social science training set. A contingency table was constructed to compare the model’s predictions for ambiguous and non-ambiguous sentences in the test set. Of the 117 sentences previously labeled as ambiguous in the full dataset, 23 were found in the test set. The model correctly classified 20 of these ambiguous sentences and misclassified 3. For non-ambiguous sentences, 198 were correctly classified, while 25 were misclassified. To evaluate the statistical significance of any difference in classification accuracy between these groups, we performed a Fisher’s exact test. The test resulted in an odds ratio of 0.842 and a 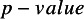 of 0.733. The 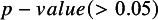 indicates no statistically significant difference in the model’s performance between ambiguous and non-ambiguous sentences. These results suggest that the model’s ability to classify causal statements was not substantially affected by the ambiguity identified during manual labeling.

The analysis of misclassified sentences can provide further insight into the model’s performance. For non-causal sentences incorrectly classified as causal (21 instances), the model appeared to be sensitive to complex sentence structures, hypothetical scenarios, and comparative language. Sentences describing experimental setups or using conditional statements were sometimes misinterpreted as causal. For example, descriptive statements about game theory concepts or hypothetical player behaviors were occasionally miscategorized. Verb analysis of these misclassified non-causal sentences showed that descriptive verbs such as “is,” “become,” and “has been” were often present in sentences flagged as causal. These verbs, while not inherently causal, may have been associated with causal relationships due to their context within explanatory or complex sentences.

Regarding causal sentences misclassified as non-causal, it is important to note the limited sample size of only five instances in our test set. This small number restricts our ability to draw broad conclusions about the model’s performance in such sentences. However, in these few cases, we observed that some sentences used indirect language to express causality. For instance, sentences employing phrases like “sensitive to” or “proposed as a means to promote” presented classification challenges. These observations indicate that certain linguistic structures common in academic writing can lead to misclassification. Complex relationships described in a single sentence, the use of conditional language, and the presence of explanatory clauses may contribute to false positives in causal classification. Conversely, the few instances of missed causal relationships suggest that more subtle or indirect expressions of causality can be overlooked.

While these findings highlight specific sentence types that may pose difficulties for the model, they should be interpreted within the context of the model’s overall strong performance. The relatively small number of misclassifications, particularly for causal sentences incorrectly labeled as non-causal, suggests that the model generally handles the complexities of causal language in social science texts effectively.

### 4.2 Summary

This study presented the development and evaluation of multiple models designed to distinguish causal from non-causal sentences in social science literature. Our approach involved fine-tuning transformer-based models on general-purpose and curated domain-specific datasets. The objective was to compare models and fine-tuning approaches to improve their applicability of text-mining techniques for causal sentences extraction within the social science domain.

The results show that the BERT model fine-tuned on social science training data achieved the best performance on the social science test set based on the F1 macro score. The study also identified a domain shift bias, as the performance of models fine-tuned on the general-purpose training set dropped by 4%–18% in F1 macro score when tested on the social science test set, compared to their performance when tested on the general-purpose training set. Conversely, when the models were fine-tuned on the social science training set and evaluated on the social science test set, their F1 macro scores increased by 7%–15%, compared to their performance when fine-tuned on the general-purpose training set, highlighting the importance of fine-tuning on domain-specific datasets for improved performance.

## 5 Discussion

### 5.1 Key findings

The results indicate that fine-tuning language models on domain-specific social science datasets enhance their performance. Models fine-tuned on the social science training set demonstrated superior performance on the social science test set compared to models fine-tuned on general-purpose datasets. Conversely, models fine-tuned on general-purpose datasets performed better on the general-purpose test set than those fine-tuned on social science data. For example, RoBERTaLarge fine-tuned on the general-purpose dataset achieved the highest F1 macro score of 0.81 on the general-purpose test set, outperforming models fine-tuned on the social science dataset. This reciprocal relationship underscores the importance of domain-specific fine-tuning and provides clear evidence for domain specificity in model performance. Social science domain is characterized by complexity and nuanced writing styles,^43^ which necessitates models capable of capturing such intricacies and nuances.

Our results suggest that the best model for causality extraction depends on the domain and task requirements. There is always a trade-off between minimizing false negatives and false positives. For example, if the primary goal is to extract the maximum number of causal sentences, the BERT model fine-tuned on the social science training set, and LLAMA2-7b fine-tuned on the general-purpose training set achieves the highest recall with a score of 0.94. Conversely, if minimizing false positives is paramount, the SciBERT model fine-tuned on the social science training set exhibits the highest precision with a score of 0.91. Ultimately, SciBERT fine-tuned on the merged dataset emerged as the optimal model for achieving balanced performance across both datasets, attaining an F1 macro score of 0.79 on the general-purpose test set and 0.85 on the social science domain test set. In addition to performance metrics, computational limitations must also be factored into model selection.

The results indicate that model complexity does not necessarily lead to better performance. Conversely, the more complex models like LLAMA2-7b, and Mistral-7b exhibited weaker performance and lower generalizability when fine-tuned on social science and general-purpose training sets. When computational resources are constrained, and model performances are comparable, LLAMA2-7b and Mistral-7b can be readily excluded from consideration. The findings underscore the importance of critical evaluation rather than relying on the assumption of scale optimism in language models.^44^ While leveraging increasingly complex models is alluring, their weaker performance suggests that model scale does not inherently equate to superior performance. The research community must prioritize human verification processes to mitigate potential inaccuracies, biases, and limitations, irrespective of model scale.^45^ To enhance reliability, integrate expert review of model outputs, particularly those classified with uncertain classifications, and implement an iterative refinement process where human feedback improves the model.^46^ When multiple reviewers are involved, assessing inter-rater reliability becomes crucial to ensure consistency. A nuanced approach is needed, where model selection is guided by performance metrics, resource constraints, and domain-specific requirements rather than assumptions of scale-driven improvements. These strategies can help researchers balance the efficiency of automated extraction with the precision required in scientific literature review, particularly given the often ambiguous nature of causal language in social sciences.

Evaluating the models’ performance separately on extracting causal and non-causal sentences in the social science test set revealed that models fine-tuned on general-purpose data exhibited a bias toward classifying most samples as causal. Across all models, the recall for the causal label was higher than precision, and consequently, the recall was lower for non-causal labels. This might indicate that general-purpose models struggle to capture the nuances of causal sentences in the social science domain. Conversely, when models were fine-tuned on the social science training set and evaluated on the general-purpose test set, all models exhibited higher scores across metrics for extracting non-causal sentences but poor performance in extracting causal sentences. This could be attributed to two potential reasons: firstly, the general-purpose test set is imbalanced, with causal samples being the minority class, causing any incorrect prediction of this label to significantly impact metric scores. This imbalance issue is evident even for models fine-tuned on the general-purpose and merged training sets and tested on the general-purpose test set. Secondly, overfitting to the domain-specific data may have occurred, resulting in a lack of generalizability. These biases were more pronounced in the more complex models like LLAMA2-7B and Mistral-7B, which have more intricate architectures, and a larger number of learnable parameters compared to RoBERTa-Large, BERT, and SciBERT.

The tendency toward overfitting appears mitigated by fine-tuning on the merged training set, comprising social science and general-purpose training data for LLAMA2-7B and Mistral-7B, exhibiting better generalizability. The gap between social science and general-purpose F1 macro scores decreased by approximately 2% for these models, without a change for LLAMA2-7B compared to fine-tuning solely on the general-purpose training set. In contrast to fine-tuning only on the social science dataset, the generalizability gap, measured by the F1 macro score, decreased by approximately 25% for LLAMA2-7B and 23% for Mistral-7B.

### 5.2 Implications

The present work addresses an important gap in the field of social science by curating a specialized dataset for causal language extraction, accompanied by a model specifically fine-tuned on this dataset. This advancement facilitates the precise extraction of causal sentences within the social science domain, enhancing the field’s methodological toolkit. Our study reproduces the domain shift bias reported by Moghimifar et al.,^14^ as the results demonstrate that general-purpose models do not generalize well to our social science benchmark dataset. Additionally, our work extends the study by Tan et al.^24^ by considering additional models beyond BERT, providing separate metrics for positive and negative labels, and various study designs by mixing social science and general-purpose training sets. Our study design provides information that allows scholars to make an informed decision when selecting a model for causal sentence extraction, prioritizing precision or recall based on their research needs. Furthermore, several of our models outperformed the status quo in terms of their accuracy in extracting causal sentences from non-causal ones in the social science domain.

This study goes beyond prior work in causality extraction, which focused on classifying causal claims in hypotheses.^47^ Causal statements can occur throughout scientific manuscripts; for instance, claims in an introduction may reflect causal assumptions based on prior research, and even when a hypothesis is tentatively phrased, causal claims in the results or discussion section may be emboldened by positive evidence. No matter their position in a manuscript—causal claims speak to the “latent theory” in a scientific discipline; they reflect what mechanisms scholars believe might be at play. By extracting sentences from full-text manuscripts, the current study has broader applicability. Moreover, using transformer models enables context handling and facilitates effective transfer learning for fine-tuning with limited data.

Building upon prior research utilizing text mining for qualitative research synthesis, such as the study by van Lissa,^10^ which demonstrated the value of inductively identifying themes and relationships within a corpus, the present work takes a more granular approach by focusing on extracting explicit causal claims across academic texts. Automating the extraction of causal claims opens new avenues for large-scale analysis and comprehensive mapping of existing theories and mechanisms across diverse social science fields to identify patterns, establish correlations, and, most importantly, explore the causal mechanisms that link social phenomena.^48^

The current study paves the way for further analysis of causal claims in the social sciences, for example, using topic modeling to uncover commonly made causal claims^49^ or using the causal networks to infer logical rules or patterns, enabling the prediction of novel events based on the potential relationships between constructs represented within these networks. The present study is a first step toward the goal of comprehensively mapping the cause-effect relationships studied in a specific body of literature—in a sense, the “latent theory” authors in that field subscribe to. Given the fact that only 15% of hypothesis-testing social science studies explicitly refer to a specific theory,^50^ many causal beliefs are implicit, and much can be gained by cataloging those implicit claims. By extracting causal sentences, the present study takes a necessary first step for any further analysis involving the identification of causes and effects within a domain, the creation of causal graphs,^51^ or further analysis over such graphs.^52^

### 5.3 Limitations

This study has several limitations to consider. First, the benchmark data originated solely from the CoDa, raising concerns about potential source bias. While CoDa is a highly curated dataset and thus an ideal sandbox example for validating new research synthesis methods, it does not represent the broader social science literature. As previously noted, a recognized limitation of automated causal sentence extraction is domain shift bias, which can also extend to sub-domains. Given the wide range of specialized research areas within social science, each with its terminology, this limitation could hinder the model’s generalizability across new sub-domains. We have shown that performance significantly improves by fine-tuning the candidate models with a limited but targeted dataset from the specific domain of interest. Our open-source code enables other researchers to fine-tune the model for their unique sub-domains with their benchmark datasets, effectively addressing domain shift biases and enhancing the model’s applicability across social science.

One important consideration is that the ambiguity encountered in social scientific causal claims might reflect the inherent complexity of causality in social phenomena. Causality might be bidirectional, indirect, or complex, and even human experts may disagree on the causal nature of certain relations, indicating that a “perfect” model performance may be theoretically unattainable. Further research needs to address this inherent ambiguity by using evaluation methods and expert consensus to determine the theoretical “upper bound” of model performance.

Another limitation is that the present method only identifies causality at the sentence level, thus potentially missing causal claims made across multiple sentences.^11^ Since identifying multi-sentence causality requires a different preprocessing approach, it is beyond the scope of the present study. Furthermore, the current work does not address the specific nature of the cause-and-effect relationships (e.g., whether the effect is positive or negative). Finally, the present study does not yet offer a way to categorize or classify causal claims. For example, the sentences “In-group identification promoted cooperation with group members” and “People in the blue group were more likely to cooperate with others in the blue group” both convey a causal link between group membership and cooperation but appear idiomatically distinct. While we acknowledge this limitation, addressing it is out of scope for the present study. Our work provides a method to curate the data needed for future work to address challenges like these.

### 5.4 Future research

The present study establishes a baseline for future research on causal claims extraction within social science literature by curating a tailored dataset and fine-tuning models for this domain. The fully reproducible nature of our code presents opportunities for future research into the performance of LLAMA 3^53^ and other emerging models and methods, such as few-shot learning, in the context of extracting causal sentences in social sciences. Furthermore, developing a more comprehensive dataset could yield improved model performance and enhance generalizability across the various disciplines and subfields within the social sciences. Given the inherent complexity of social science, the observed superior performance of models on the social science dataset compared to the general-purpose dataset is noteworthy. This phenomenon, coupled with findings that larger models underperform smaller ones in causal sentence extraction, merits further investigation. Future research should explore the underlying factors contributing to these results, offering insights into the interplay between model architecture, dataset characteristics, and domain-specific challenges.

Text mining can contribute to inductive research by identifying the hypothesized directionality and mechanisms implied by relationships between phenomena by applying methods that map cause-effect networks. Providing an initial causal framework for inductive reasoning would facilitate theory development. Future work could take the current approach further and map associations between constructs in a network.^51^ Building upon this network approach, future research could explore the construction of Directed Acyclic Graphs (DAGs) to represent the complex web of causal relationships extracted from the literature.^54^ These DAGs could be organized hierarchically based on specificity, domain, or empirical support, facilitating a structured and navigable catalog of causal claims.

Our work lays the foundation for future software tools that can assess, and flag ambiguous causal claims made by authors, much like how existing programs such as Statcheck^55^ automatically detect statistical reporting inconsistencies. Such software could assist authors, reviewers, and editors by monitoring and improving the explicitness and accuracy of causal claims in social science research. For instance, our method could screen manuscripts for causal claims in the introduction, check their congruence with model specifications in the methods or analysis sections, and verify that discussions are consistent with the results. This assists in identifying causal claims that are assumed without proper design, thereby improving research integrity.

Automated methods could group similar claims to manage the abundance of extracted claims, enabling meta-analyses that provide aggregated insights and highlight areas of consensus or disagreement. Temporal tracking of claims could illuminate the evolution of causal theories over time while classifying claims based on the strength of supporting evidence would help prioritize well-substantiated relationships. Integrating this catalog with databases of empirical studies and their effect sizes could offer a more comprehensive view of the evidence supporting each causal claim. These advanced techniques for organizing and analyzing extracted causal information could significantly enhance our ability to synthesize knowledge across the social sciences, potentially leading to new theoretical insights and research directions.

## 6 Conclusions

This study advances the automatic extraction of causal sentences from social science literature. We created a specialized dataset and fine-tuned transformer models to address the challenge of complex causal language in social sciences. Our main finding highlights the crucial role of domain-specific fine-tuning. Models fine-tuned on general-purpose data performed poorly in the social science context. In contrast, those fine-tuned on our social science dataset showed significantly better performance, overcoming the issue of domain shift bias. This approach improved the accuracy of extracting causal sentences from social science papers, even with limited training data. Interestingly, the results also showed that more complex models did not necessarily lead to better performance, emphasizing the need for critical evaluation rather than relying solely on assumptions of scale-driven improvements.

The provided open-source dataset and fine-tuned models in this work lay the foundation for comprehensive, large-scale analysis of causal claims across the social sciences. By systematically cataloging existing causal statements, this research enables the exploration of the mechanisms underlying social phenomena, supports theory development, and strengthens the methodological rigor of the field. Future work can leverage these resources to uncover patterns in causal reasoning and further advance our understanding of the social world.

## Data Availability

The dataset and code for this study are available at https://github.com/rasoulnorouzi/cessc under the GNU GPLv3 license.
